# PET segmentation of bulky tumors: Strategies and workflows to improve inter-observer variability

**DOI:** 10.1371/journal.pone.0230901

**Published:** 2020-03-30

**Authors:** Elisabeth Pfaehler, Coreline Burggraaff, Gem Kramer, Josée Zijlstra, Otto S. Hoekstra, Mathilde Jalving, Walter Noordzij, Adrienne H. Brouwers, Marc G. Stevenson, Johan de Jong, Ronald Boellaard

**Affiliations:** 1 Nuclear Medicine and Molecular Imaging, University of Groningen, University Medical Center Groningen, Groningen, The Netherlands; 2 Department of Radiology and Nuclear Medicine, Cancer Center Amsterdam, Amsterdam, The Netherlands; 3 Department of Oncology Medicine, University of Groningen, University Medical Center Groningen, Groningen, The Netherlands; 4 Department of Surgical Oncology, University of Groningen, University Medical Center Groningen, Groningen, The Netherlands; Chongqing University, CHINA

## Abstract

**Background:**

PET-based tumor delineation is an error prone and labor intensive part of image analysis. Especially for patients with advanced disease showing bulky tumor FDG load, segmentations are challenging. Reducing the amount of user-interaction in the segmentation might help to facilitate segmentation tasks especially when labeling bulky and complex tumors. Therefore, this study reports on segmentation workflows/strategies that may reduce the inter-observer variability for large tumors with complex shapes with different levels of user-interaction.

**Methods:**

Twenty PET images of bulky tumors were delineated independently by six observers using four strategies: (I) manual, (II) interactive threshold-based, (III) interactive threshold-based segmentation with the additional presentation of the PET-gradient image and (IV) the selection of the most reasonable result out of four established semi-automatic segmentation algorithms (Select-the-best approach). The segmentations were compared using Jaccard coefficients (JC) and percentage volume differences. To obtain a reference standard, a majority vote (MV) segmentation was calculated including all segmentations of experienced observers. Performed and MV segmentations were compared regarding positive predictive value (PPV), sensitivity (SE), and percentage volume differences.

**Results:**

The results show that with decreasing user-interaction the inter-observer variability decreases. JC values and percentage volume differences of Select-the-best and a workflow including gradient information were significantly better than the measurements of the other segmentation strategies (p-value<0.01). Interactive threshold-based and manual segmentations also result in significant lower and more variable PPV/SE values when compared with the MV segmentation.

**Conclusions:**

FDG PET segmentations of bulky tumors using strategies with lower user-interaction showed less inter-observer variability. None of the methods led to good results in all cases, but use of either the gradient or the Select-the-best workflow did outperform the other strategies tested and may be a good candidate for fast and reliable labeling of bulky and heterogeneous tumors.

## Introduction

In oncology, Positron Emission Tomography combined with Computed Tomography (PET/CT) using the tracer fluorodeoxyglucose (FDG) is important for cancer diagnosis [1–3]. In order to assess tumor staging and response to therapy, the most commonly used measurements are the maximum Standardized Uptake Value (SUV_MAX_), the mean SUV (SUV_MEAN_), and total lesion glycolysis (TLG) which is defined as tumor volume times SUV_MEAN_, which are extracted from the segmented tumor. Recently, features containing more detailed information about tumor phenotype and intra-tumor heterogeneity have been reported. Previous studies demonstrated the clinical relevance of these feature values [4–6]. Especially for patients with advanced stage cancer with bulky tumors, analysis and evaluation of these feature values can add valuable information and help to direct treatment.

Since these features are highly sensitive to tumor delineation [5,7], a reliable and reproducible segmentation is essential. For this purpose, a segmentation strategy with low inter-observer variability is important. Due to patient motion, image noise, and varying intrinsic contrast, the tumor borders are not clearly defined in a PET image, which makes a segmentation challenging [8]. Up to now, tumors are still mainly segmented manually what is time-consuming, subjective, and leads to a high inter-observer variability [9–11]. One important aspect influencing manual segmentation performance is that the tumor appearance depends on the intensity window used for displaying the image. This intensity window can be changed by the observer and changes the tumor appearance (i.e. makes the tumor to appear bigger or smaller) in the visualization due to the partial volume effect. Especially for large tumors (metabolic active tumor volume (MATV) > 300mL) with irregular and complex shapes, a manual segmentation is very time consuming and prone to segmentation errors.

In order to facilitate the segmentation task, several automatic segmentation algorithms have been developed. Some methods use simple thresholding, defining all values above a percentage value of SUV_MAX_ or a fixed SUV (usually 4 or 2.5) as tumor [12]. Other adaptive thresholding techniques take into account the tumor-to-background ratio or the object size [13,14]. Furthermore, segmentation approaches using advanced stochastic techniques or machine learning algorithms have been proposed and evaluated, showing good results for both phantom and patient studies [15]. However, the majority of these approaches are not publicly available and have only been tested on specific datasets. Moreover, none of these methods is used in clinical practice, as all of them have limitations.

It is important to note that especially for large heterogeneous bulky tumors, a user-interaction step will remain necessary in order to get a valid and plausible segmentation as one (semi-) automatic segmentation method is unlikely to provide good results in all cases [16]. In order to illustrate the special challenges coming with complex tumors, we evaluated three automatic segmentation algorithms and applied them on the dataset used in this study. The results are displayed in the S1 Material. As can be seen, none of the automatic segmentation algorithms was able to properly segment all tumors. In order to reduce the inter-observer variability and to overcome the limitations of automatic segmentation algorithms, it might be advantageous to reduce the user-interaction in the segmentation process without making the segmentation fully automatic.

For this purpose, three new segmentation workflows were evaluated in this study aiming to reduce user-interaction and thereby potentially improving inter-observer variability. In the first introduced workflow the user is asked to change the percentage of the SUV_MAX_ threshold interactively until a satisfactory segmentation is achieved. I.e. the user adapts the boundary of the segmentation by only changing the threshold using an interactive slider rather than the common use of a fixed predefined threshold value. The second strategy is inspired by the automatic gradient-based segmentation approaches: the observer was presented with both the PET-intensity as well as the PET-gradient image, highlighting tumor boundaries. Next, the user was asked to change the percentage of the SUV_MAX_ threshold interactively as described above. This workflow was implemented in order to mitigate the effect of the chosen intensity window on the segmentation outcome as the gradient image displays the tumor boundaries independent of the intensity window. In the last new workflow, the user needed to select the preferred result from four predefined segmentations based on four widely known delineation algorithms.

These strategies are especially suited for the segmentation of bulky tumors, e.g. for the use of MATV as prognostic factor in lymphoma patients or to use metabolic information to measure treatment response [17]. Furthermore, the strategies can, for example, also be used for the fast generation of reliable training sets for Convolutional Neural Networks (CNN) which are used more and more frequently for segmentation tasks [18–20]. The aim of this study was to investigate the potential improvements in the inter-observer variability of tumor segmentation results using these new workflows compared with more standard segmentation approaches, while allowing for the generation of plausible and reliable segmentations. The strategies were applied on patients with advanced oncological diseases suffering from especially large and heterogeneous tumors, being the most challenging cases for which traditional workflows fail.

## Materials and methods

**Fig 1:**
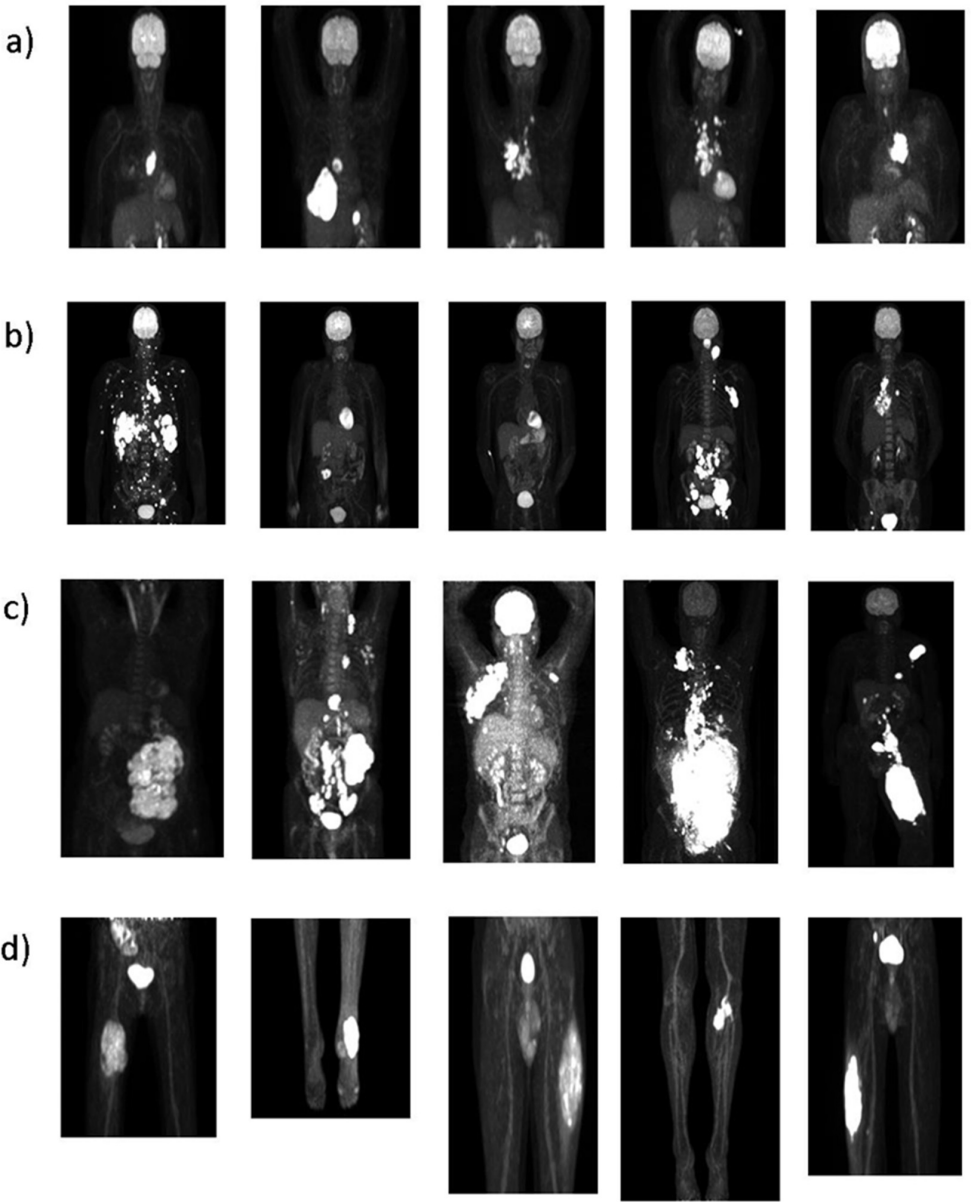
MIP of every patient included in the study ordered by tumor type: a) lung cancer, b) lymphoma, c) melanoma, d) sarcoma.

This study has been approved by the Institutional Review Board (IRB), and the need for written informed consent was waived (IRB case number 2016.984) as well as by the Medical Ethics Review Committee of the VUMC and registered in the Dutch trial register (trialregister.nl, NTR3508). Data were collected as part of several ongoing and past studies and all patients gave informed consent for study participation and use of their data for (retrospective) scientific research. Twenty datasets of patients with stage III or IV cancer were included in this study. The patients suffered from four cancer types (five patients each): Non-Small-Cell-Lung-Cancer (NSCLC), High-grade lymphoma, melanoma and locally advanced extremity soft tissue sarcoma. Sarcoma and NSCLC patients were included in previous studies [21–23]. These studies were chosen to assure that we would have a wide range of tumor sizes, shapes, locations and uptake distributions allowing us to determine a segmentation strategy that would work best in a large ranges of bulky tumors. The scans were performed at two institutes. Melanoma and sarcoma patients were scanned on a Siemens Biograph mCT64 and the images were iteratively reconstructed using the vendor provided PSF+TOF reconstruction method with three iterations and 21 subsets (PSF+TOF 3i21s) and a post-reconstruction smoothing with a 6.5 mm full-width-at-half-maximum Gaussian kernel. Images were reconstructed to a voxel size of 3.1819 mm x 3.1819 mm x 2 mm. NSCLC and lymphoma images were acquired on a Philips Gemini TF/TOF scanner and reconstructed using the BLOB-OS-TF reconstruction with 6.5 mm full-width-at-half-maximum pre-reconstruction smoothing. All these images yielded a voxel-size of 4 x 4 x 4 mm. All images were converted from Becquerel/ml to SUV as it is commonly done in PET image analysis. SUV is calculated as the ratio of the activity concentration displayed in the image and the injected activity divided by the patient weight. A conversion of the image to SUV is beneficial as it removes variability coming with differences in patient size and injected FDG activity across images. All twenty PET images contain comparable image statistics and quality as they are EARL compliant. The maximum intensity projection of every patient is displayed in Fig 1. The corresponding patient information such as weight and injected dose can be found in the S1 Material (S1 Table).

All tumors were delineated independently by six observers with different levels of experience blinded by each other: Two experienced nuclear physicians (more than ten years of experience), one experienced medical physicist (more than twenty years of experience) and three observers with less than three years of experience in tumor delineation.

All segmentations were performed using an in-house software developed for the analysis of PET images, already used and described in previous studies [22,24,25]. The software allows the user to delineate volume-of-interests (VOI) using various segmentation techniques. The default intensity window setting displayed SUV in the range from 0–10. Yet, the observers were allowed to change the intensity window as is also often done in clinical practice. Before the start of the experiment, every tumor region was manually marked roughly with a mask. PET and corresponding low-dose CT images containing this mask were presented to the observers simultaneously (S1 Fig). Subsequently, every observer delineated the images using four strategies:

### Manual segmentation

The first segmentation was performed manually. Therefore, it was permitted to shrink the predefined mask to a smaller size using a percentage threshold of the SUV_MAX_. The percentage threshold was set by each observer individually per lesion. All voxels with an intensity value above this threshold were included in the segmented volume. The observers manually modified this segmentation by adding or deleting voxels.

### Interactive threshold-based segmentation

**Fig 2:**
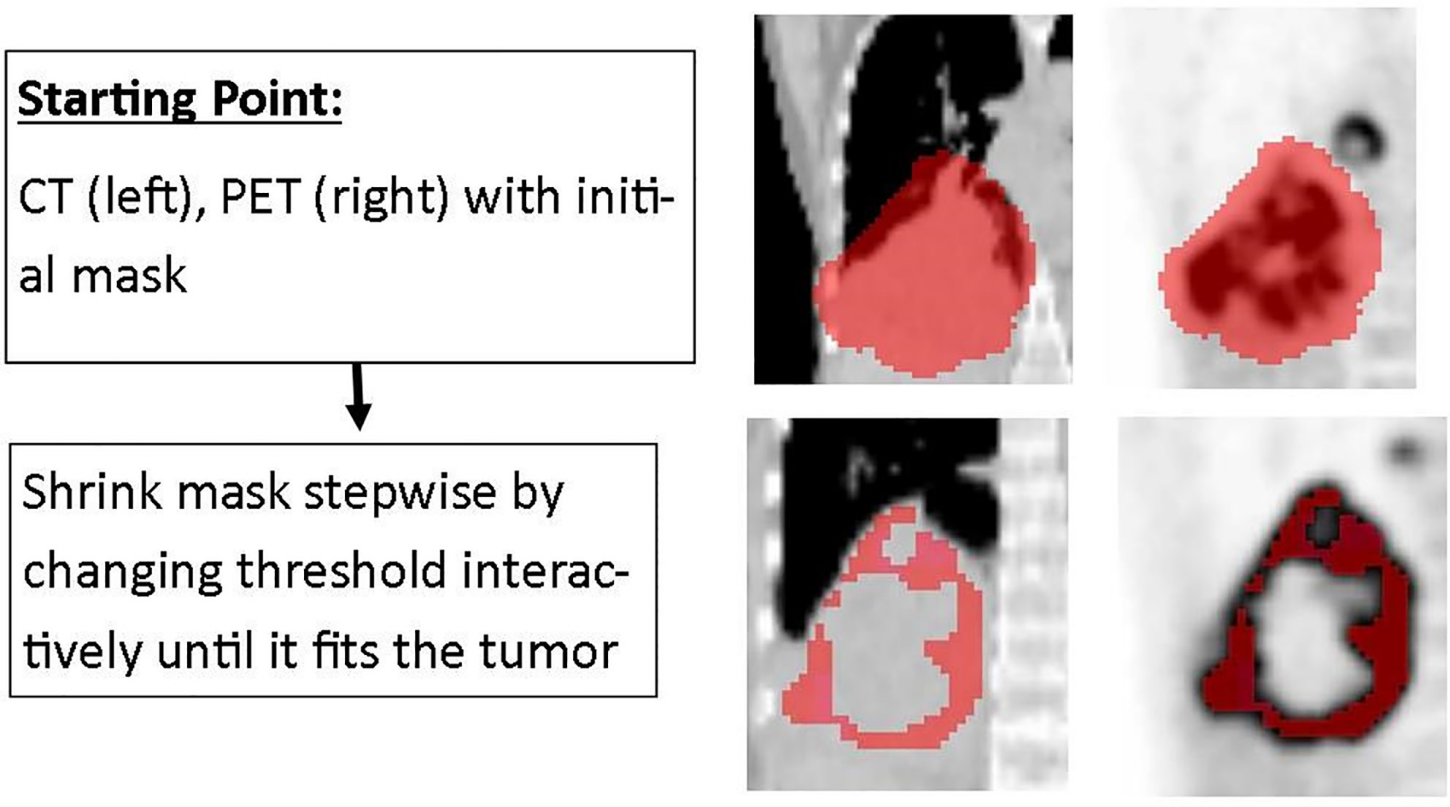
Illustrates the workflow for the interactive threshold approach. Initially, CT and PET image are presented to the user including a mask marking roughly the tumor. The user changes then interactively the threshold until the segmentation is considered as satisfactory.

Secondly, an interactive threshold-based segmentation was evaluated which was restricted to the inside of the predefined mask. The user changed the percentage threshold value (range from 0–100%) of the SUV_MAX_ interactively (as described above) until the segmentation was considered satisfactory on visual inspection This workflow is illustrated in Fig 2.

### Threshold-based segmentation including a gradient image

**Fig 3:**
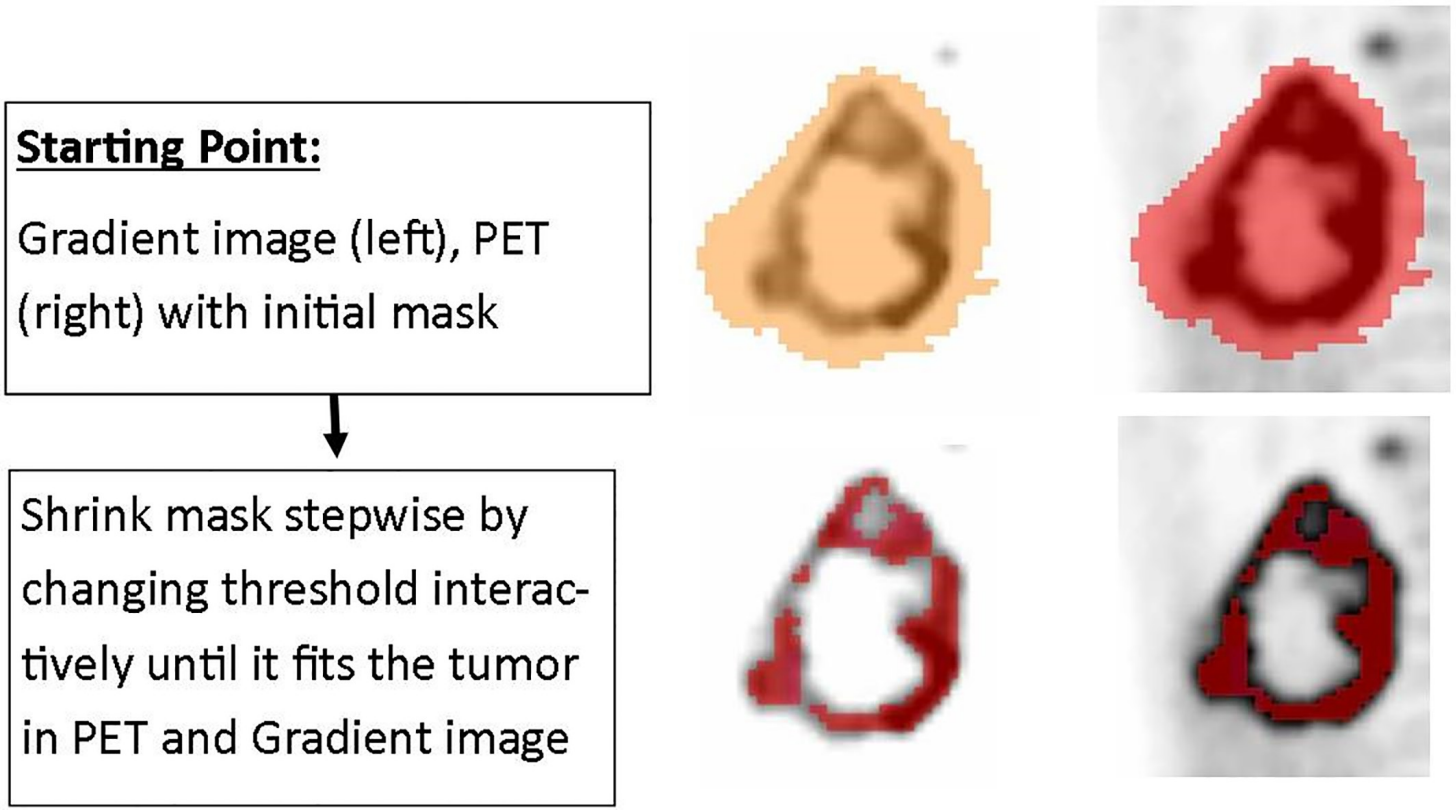
Illustrates the workflow of the interactive gradient based segmentation. Gradient and PET image are presented to the user. Also here, the user changes interactively the threshold until the segmentation is satisfactory on both PET and gradient image.

Next, the same interactive threshold-based approach was used but this time, the presented CT-image was replaced by the PET-gradient image that emphasizes the boundaries of the high-uptake regions. The user was asked to set the percentage threshold so that the border of the VOI collided with the borders pronounced in the gradient image. In the gradient image, the tumor boundaries are displayed independent of the intensity window set by the observer (see Fig 3). Therefore, this workflow was chosen in order to mitigate the possible effects of using different intensity windows by the observers on the segmentation results.

### Selection of the best result from four automatic segmentation algorithm

**Fig 4:**
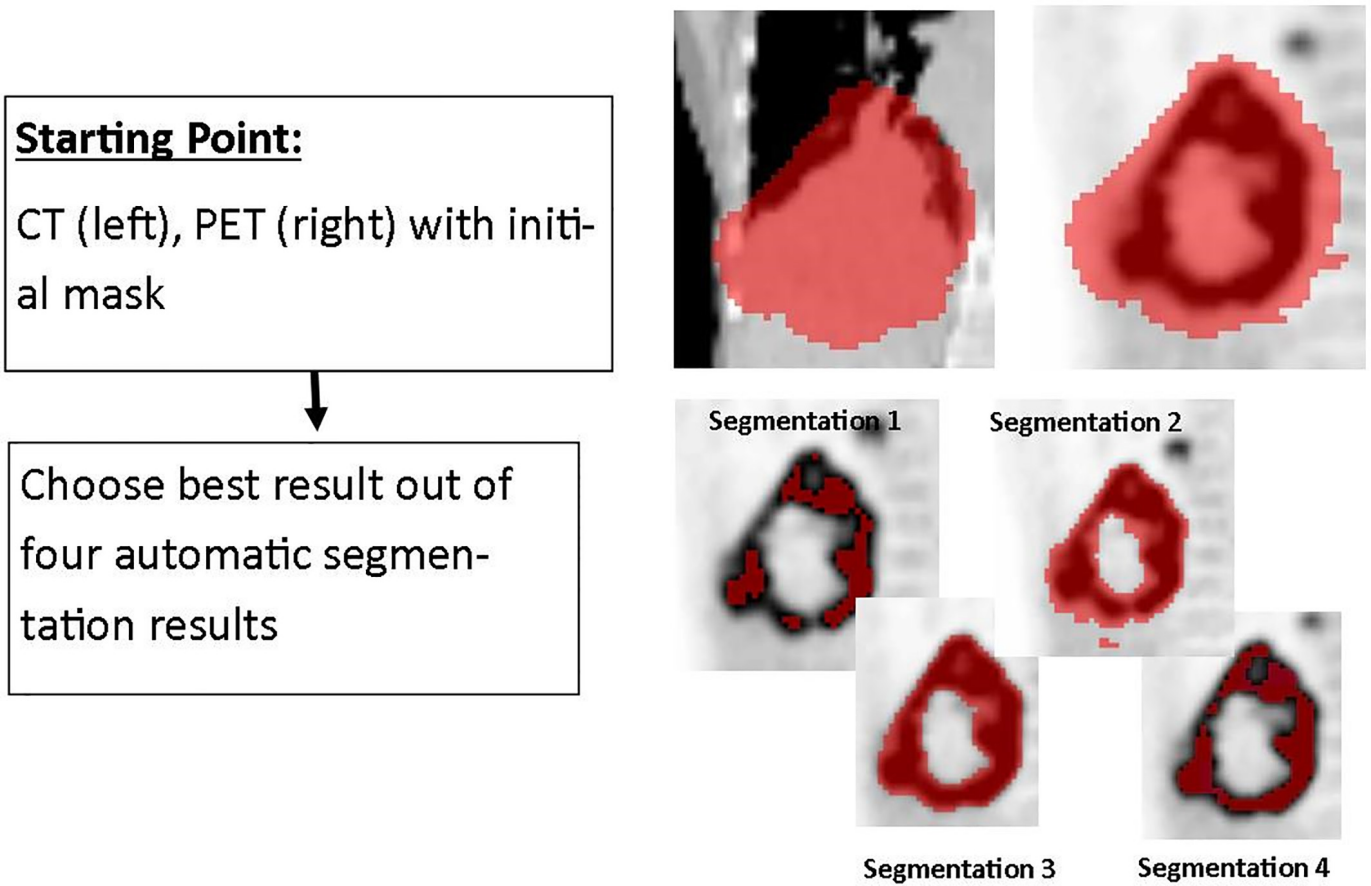
Displays an example for the Select-the-best method. The user chooses the best result out of four segmentations that were acquired automatically.

Finally, low-dose CT and PET image containing the results of four automatic threshold-based segmentation algorithms were presented to the user. All four algorithms are commonly used and established in the literature [24,26,27]. From these segmentations, the user selected the segmentation that resembled the tumor boundary best in his/her opinion. An example is illustrated in Fig 4. The segmentations of the following algorithms were presented to the observers:

- 41% SUV_MAX_: Voxels yielding a SUV higher than 41% of the SUV_MAX_
- SUV4: Voxels with a SUV higher than 4
- SUV2.5: Voxels with a SUV higher than 2.5
- AUTO: All voxels with a SUV value higher than 50% of the SUV_PEAK_ with local background correction are included in the segmentation (i.e. a contrast oriented/adapted method). For the calculation of the SUV_PEAK_, a spherical neighborhood of 1 mL (1.2 cm diameter) is defined for each voxel conform the specifications in the EANM and UPICT guidelines [28,29]. The highest mean value of all neighborhoods is defined as SUV_PEAK_.

The segmentation workflows were performed in the order listed above. By following this order, every new applied segmentation strategy required less user-interaction than the previous one.

### Data analysis

Data analysis and figure visualization were performed in Python 3.6.3 using the packages numPy, sciPy [30], and matplotlib [31].

### Inter-observer variability

The Jaccard Coefficient (JC) is a measurement for the agreement of two sets A and B and is defined as:

$${JC}_{A;B}=\frac{\left|A\cap B\right|}{\left|A\cup B\right|}$$

A JC of 1 represents perfect agreement. For every segmentation approach, the JC was calculated for all possible combinations of segmentations performed by the observers.

Furthermore, in order to assess size similarity, the percentage MATV differences were calculated. The approach with the lowest inter-observer variability was determined by evaluating the JC and MATV difference values with the Kruskal-Wallis test. The Kruskal-Wallis test ranks JC and MATV values of all approaches together. These ranks are then compared across approaches. In this way, the approach with the lowest inter-observer variability is determined not only based on the lowest mean or median value as the ranking of all JC/MATV values is taken into account. The Benjamini-Hochberg procedure with a false discovery rate of 10% is applied in order to correct for multiple comparisons.

### Majority vote comparison

A problem in the evaluation of segmentation algorithms is that in the majority of the cases no ground truth exists. Therefore, in order to obtain a reference segmentation, a majority vote segmentation (MV) was calculated for every image as it has been shown that a MV segmentation represents a reliable segmentation [32]. A MV compares segmentations of the same object and regards the voxels marked by more than half of the segmentations as part of the VOI [33]. All other voxels are considered as segmentation error. The segmentations performed by the three experienced observers were included in the calculation of the MV segmentation. Moreover, for comparison, a MV segmentation including the segmentations of all six observers was calculated as well. All MV segmentations were visually checked for plausibility.

Reference and performed segmentations were compared regarding their sensitivity (SE) and positive predictive value (PPV). PPV and SE also measure the agreement of two sets, considering one set as reference standard [34]. Hence, SE and PPV include knowledge about voxels which are incorrectly not included (false negatives (FN)) or incorrectly included (false positives (FP)) in the comparable segmentation [34]. SE of set A and reference standard B is defined as ratio between number of voxels correctly included in the segmentation (true positives (TP)) and number of voxels of set A:

$${SE}_{A;B}=\frac{\left|TP\right|}{\left|TP|+|FN\right|}=\frac{\left|A\cap B\right|}{\left|A\right|}$$

While PPV is defined as ratio of numbers of TP and sum of number of voxels of TP and FP:

$${PPV}_{A;B}=\frac{\left|TP\right|}{\left|TP|+|FP\right|}=\frac{\left|A\cap B\right|}{\left|B\right|}$$

PPV and SE values are often combined in one value as a weighted sum. The sum weights depend on the purpose of the segmentation. In our case, in order to combine both measurements in a single value, the mean of both values was calculated:

$$PPV/SE=\frac{SE+PPV}{2}$$

PPV/SE values were calculated per tumor. Moreover, percentage MATV differences were calculated between MV and every performed segmentation. For every image, inter-observer differences and range of both metrics were compared across approaches using the Kruskal-Wallis test as explained above. In order to assess the influence of user experience, percentage MATV differences were compared between observers using the Wilcoxon signed rank test.

### Feature value comparison

To measure the variability of feature values across segmentations, percentage feature differences of performed and MV segmentation were calculated. In this study, the focus lies on the most frequently reported and most established features: SUV_MAX_, SUV_MEAN_, and TLG. Also here, variability and range of percentage differences were compared across approaches.

### Select-the-best evaluation

Fixed threshold-based segmentation methods are often used as standard approach in clinical practice, but none of them are able to generate proper segmentations in all cases and often fail in case of large heterogeneous tumors. Yet, we will report how often the result of one of the 4 automatic methods was regarded as best segmentation in the Select-the-best-approach.

## Results

### Inter-observer variability

**Fig 5:**
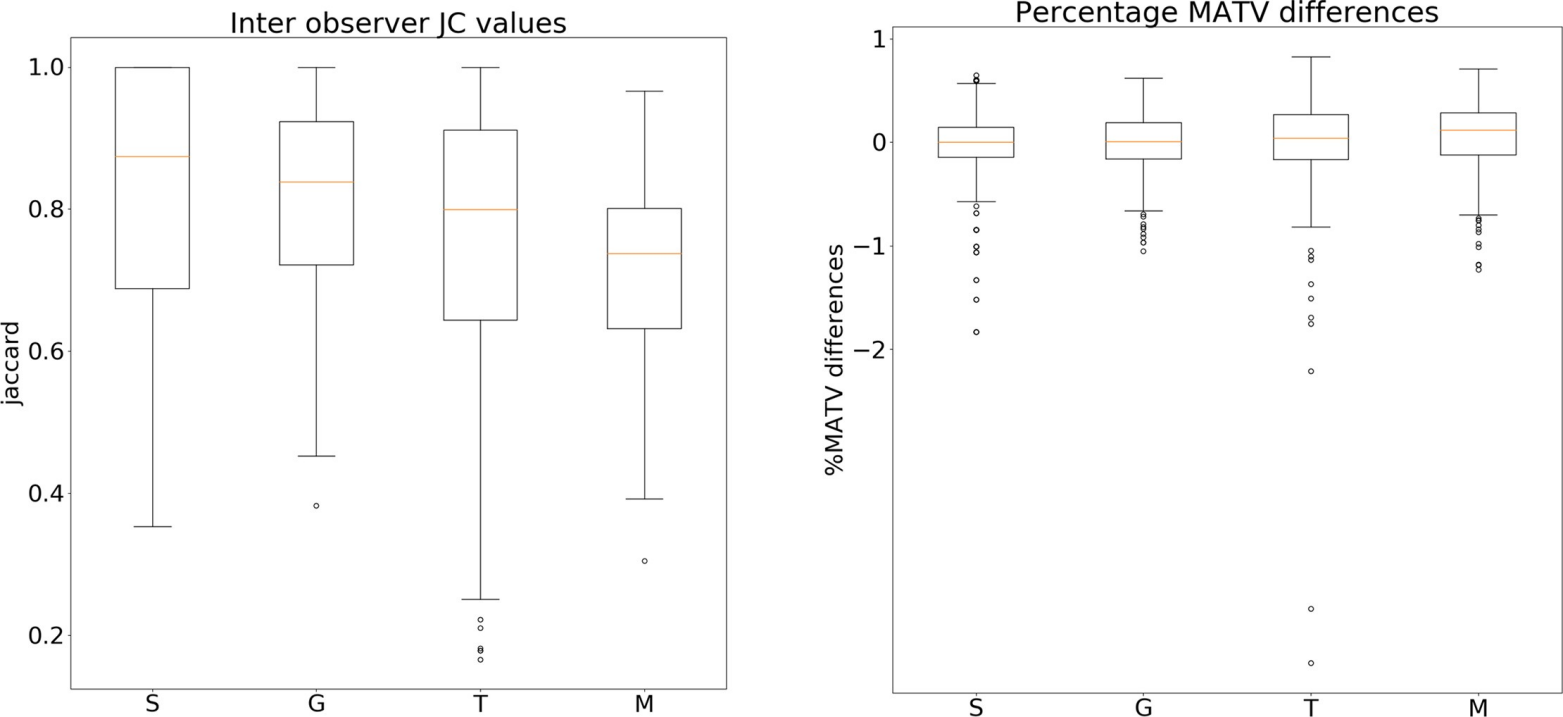
Illustrates the variability of the JC values (left) and percentage MATV differences (right) for all images. The amount of user-interaction increases from left to right (for both plots: left: Select-the-best (S), middle-left: Gradient (G); middle-right: Threshold-based (T), right: Manual (M)).

The variability of JC values and percentage MATV differences are demonstrated in Fig 5. With increasing user-interaction the variability of both metrics increases. Median and third quartile JC values are the highest, while median and IQR of percentage MATV differences are lowest for select-the-best, followed by gradient, interactive threshold and manual approach. All median, quartile values and IQR are listed in S2 Table.

A comparison between the strategies using the Kruskal-Wallis test showed that JC and percentage MATV differences of select-the-best and gradient based strategies are significantly different than the values of the other two strategies (p-value<0.01). While select-the-best and gradient, as well as interactive threshold-based and manual segmentations show no significant differences when compared with each other (see Table 1).

**Table 1:** P-values obtained with the Kruskal-Wallis test. Non-significant results are marked with ‘n.s.‘.

	All images JC	All images percentage MATV
Select-the-best vs. Gradient	n.s.	n.s.
Select-the-best vs. Threshold	<0.01	<0.01
Select-the-best vs. Manual	<0.01	<0.01
Gradient vs. Threshold	<0.01	n.s.
Gradient vs. Manual	<0.01	<0.01
Threshold vs. Manual	n.s.	<0.01

### Majority vote comparison

**Fig 6:**
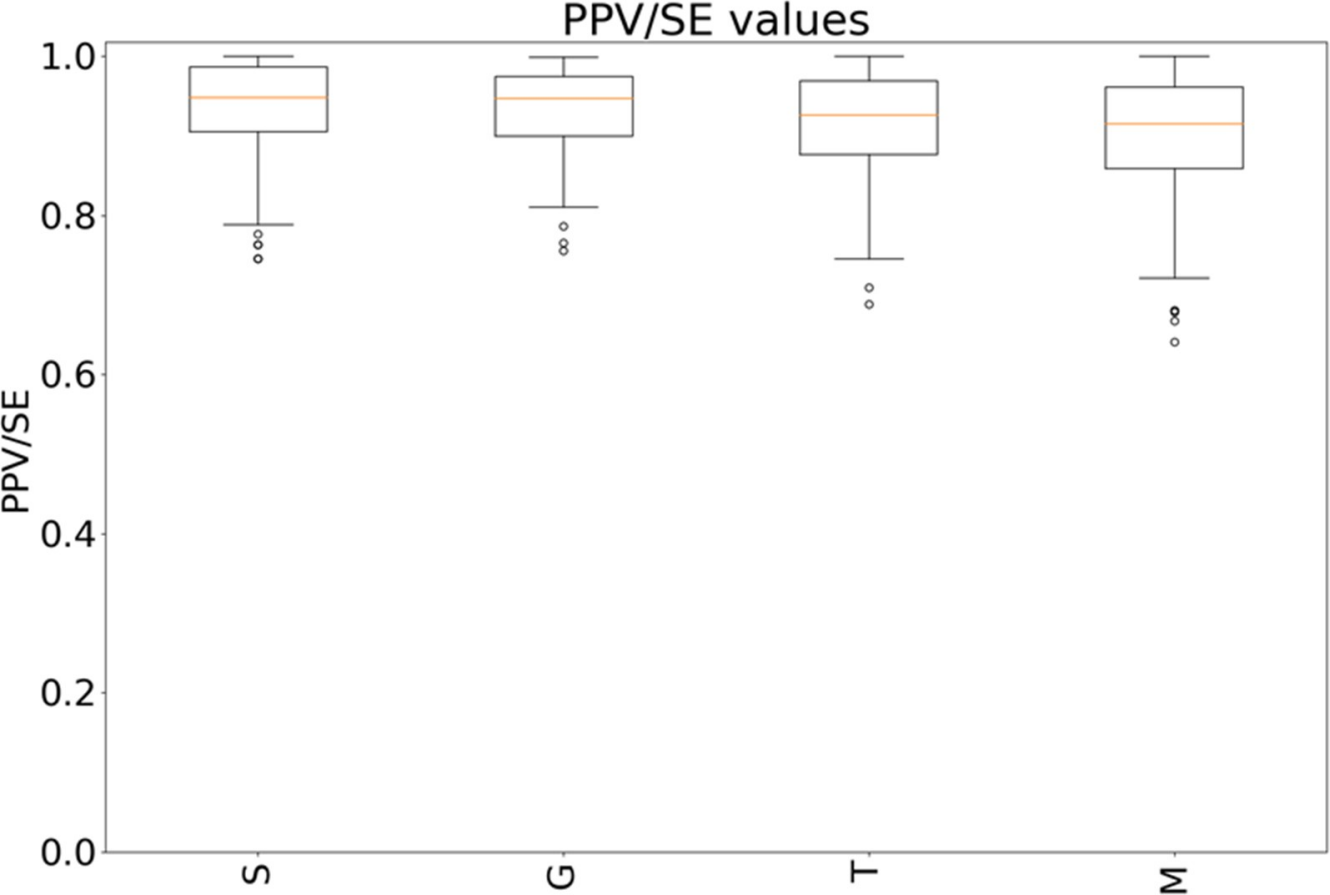
Illustrates the variability of PPV/SE values for the approaches with increasing user-interaction from left to right.

Fig 6 illustrates the variability of PPV/SE values of performed and MV reference segmentation. Select-the-best and gradient workflow result in similar values with slightly higher values for select-the-best method (Select-the-best: IQR: 0.91–0.99; Gradient: IQR: 0.90–0.97). The differences between these and the other two strategies are more pronounced (Threshold-based: IQR: 0.88–0.97; Manual: IQR: 0.86–0.92). The higher values of Select-the-best and Gradient strategy support the hypothesis that these two strategies lead to more reliable segmentations.

**Fig 7:**
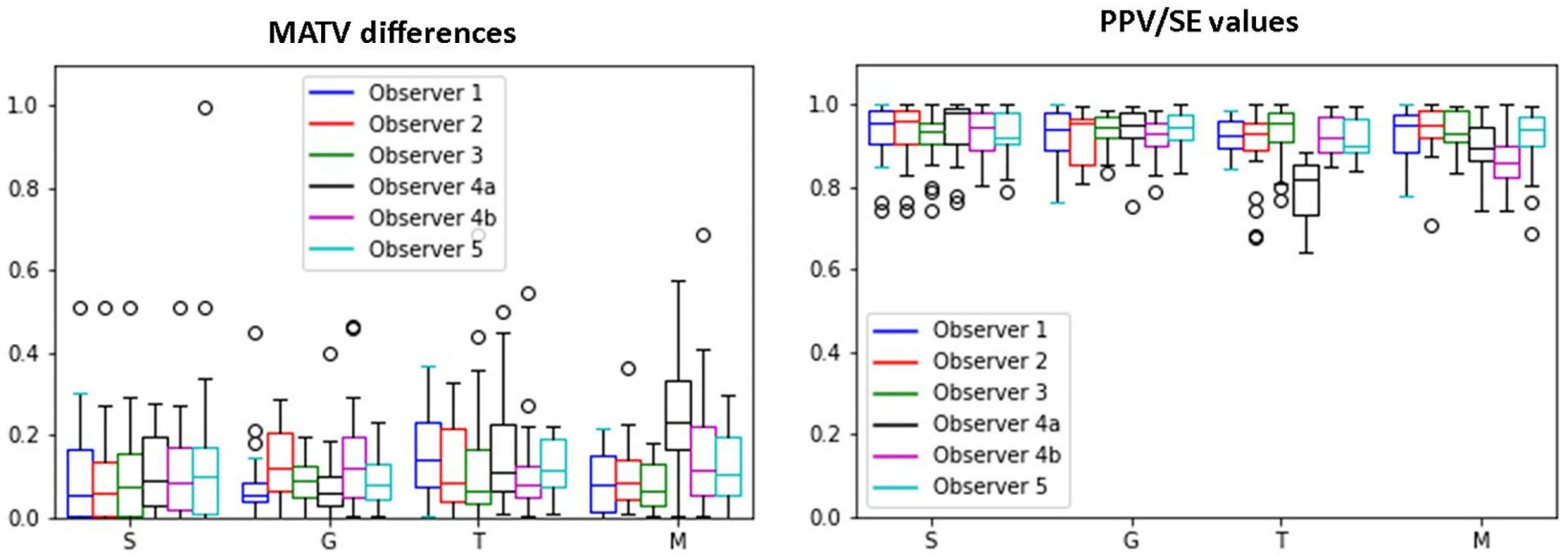
Percentage MATV differences and PPV/SE values between segmentations performed by observers and MV segmentation displayed for every observer separately. The observers are ordered by their level of experience with observer 1 being the most experienced. Observer 4a and 4b are having the same experience level.

Fig 7 illustrates the percentage MATV differences as well as the PPV/SE values of performed and reference segmentations for every observer separately. Observers are ordered according to their experience level, with observer 1 being the most experienced. All segmentation strategies show significantly lower percentage MATV differences than the manual segmentation. Also Select-the-best and interactive threshold-based segmentation result in significant differences (p-value<0.01).

Comparing percentage MATV differences and PPV/SE values between observers showed no significant differences with exception of the manual segmentation. For this method, two less experienced observers (observer 4a and 4b) showed a significant worse performance than the other observers (p-value<0.01).

Performing the same comparisons with the MV segmentation including the segmentations of experienced and less experienced observers had almost no influence on the results. Some values changed slightly but the overall findings were the same.

### Feature value comparison

**Fig 8:**
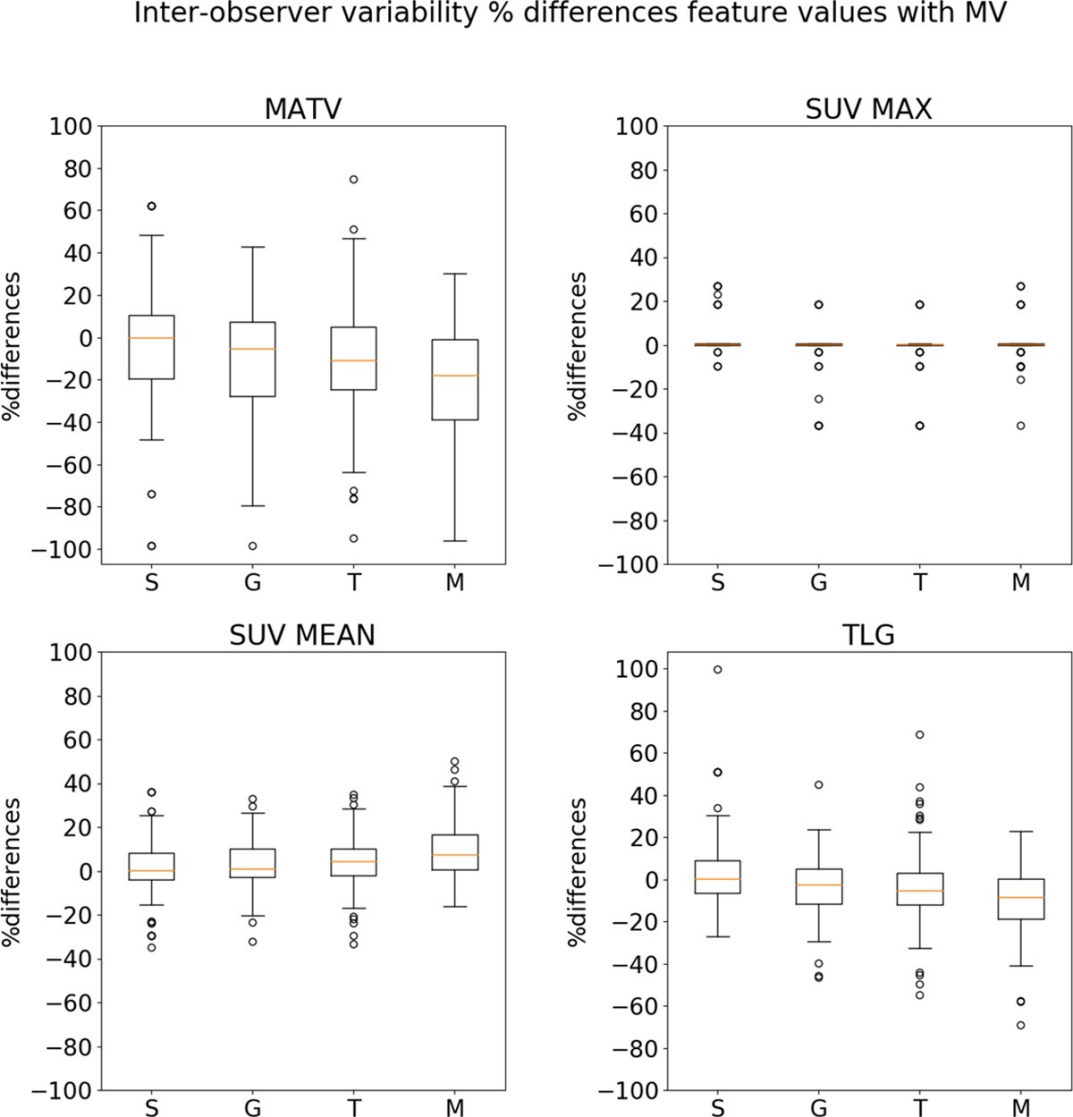
Demonstrates the feature value variability for the approaches (increasing user-interaction from left to right).

The variability of percentage differences of MATV, SUV_MAX_, SUV_MEAN_, and TLG is plotted in Fig 8. Regarding percentage MATV differences, the gradient workflow leads to the lowest IQR and median, followed by select-the-best segmentations. Interactive threshold-based and manual segmentations result in higher IQR and lower median values (S3 Table). Significant differences in percentage MATV differences were observed between select-the-best and threshold strategy, as well as between all segmentation workflows and the manual segmentations (p-value<0.01).

In the majority of the cases, the SUV_MAX_ yielded percentage differences of 0. However, the boxplot is missing four outliers of manual segmentations of one Lymphoma patient (Lympho3) which had percentage differences of more than -100% (-292.5%, -212.5%, -270.6%, -292.5%). Small discrepancies were furthermore observed for manual and select-the-best method in one Melanoma patient (Mela4) and for all approaches in another Melanoma image (Mela1). The differences between the different strategies were not significant.

SUV_MEAN_ and TLG values resulted in the lowest IQR for gradient followed by select-the-best, threshold and manual segmentations, respectively (S3 Table). Significant differences in TLG values were observed for select-the-best and all other segmentation strategies, as well as for gradient and manual segmentations (p-value<0.01). Regarding the SUV_MEAN_, all proposed workflows showed significant different values from the manual segmentation (p-value<0.01).

### Select-the-best-comparison

The SUV4 segmentation algorithm was most often considered as the best segmentation with 43 most preferred scores (35.8%). The second most chosen algorithm was the 41MAX method which was chosen 30 times (25%) as best performing segmentation. The SUV2.5 and AUTO approaches were considered 24 (20%) and 23 times (19.2%) as best.

## Discussion

In this study, we report on the inter-observer variability of four segmentation strategies especially chosen for the segmentation of bulky tumors, each of them requiring a different level of user-interaction. Our results show that the inter-observer variability improves with less user-interaction in the segmentation process. Moreover, two of the proposed strategies, i.e. using gradient information and/or predefined segmentations, seem to improve inter-observer variability compared to more conventional approaches in most cases while still generating plausible segmentations (as assessed by the observers). The proposed workflows did not only improve inter-observer variability, they also allowed a much fastersegmentation process. For the complex tumors included in the study, a manual segmentation took between twenty to forty minutes per lesion, while the interactive threshold based approach required approximately half of this time. Gradient and select the best method took less than ten and five minutes, respectively.

There might still be cases when the proposed strategies will fail. Mainly, this will be the case when the tumor is located close to another high-uptake region such as e.g. the heart. In these cases, the proposed segmentation strategies could still be used as a starting point and as second step be manually adjusted. However, in the current dataset that only happened once and therefore does not affect the overall conclusion of the paper. As the strategies improve the inter-observer variability, also additional manual adaptation of the initial segmentations should result in more reliable and reproducible segmentations, As it has been shown that the adjustment of a (semi-) automatic segmentation is more reliable that a fully manual segmentation, the results will still be preferable over a fully manual segmentation [35].

The use of an initial automated segmentation as starting point could also be the reason why the differences between manual and the interactive threshold-based strategy were not significant even if the fully interactive threshold-based approach requires less user-interaction. As for the manual segmention, the user was first allowed to shrink the tumor mask and adapted the segmentation afterwards. However, manual segmentations showed still the poorest performance in the majority of the cases and led to a high inter-observer variability consistent with finding in other previous studies [11,16].

Although manual segmentations are still considered as ground truth, it has been demonstrated that they result in less repeatable segmentation results than (semi-) automatic segmentations [36]. Repeatability of PET-based segmentations is a very important point as MATV is a metric which is frequently used for the evaluation of treatment response [24]. It is of outermost importance that changes in segmented volume are due to changes in the underlying biological tissue and not to differences in segmentation results. For this purpose, several studies indicated that segmentation accuracy is less important than repeatability [37,38] what pronounces the limitations of manual segmentations.

Shepherd et *al*. compared previously thirty segmentation algorithm with different levels of user-interaction and reported the best segmentation results for the algorithm with the highest amount of user-interaction [39]. However, the dataset used in their study had some limitations as they only included seven volumes extracted from phantom images and two patient datasets. For the dataset of our study, including only tumors with large volumes, heterogeneous uptakes and complex shapes, manual delineations were extremely labor intensive and suffered from a high observer variability. This may be explained by the profound different tumors used in our study.

Segmentations were performed by users with different levels of experience. Significant differences between experienced and less experienced observers were only observed for manual segmentations. In this case, two less experienced observers showed significantly higher percentage MATV differences and lower PPV/SE values when compared with experienced observers. This is in line with Giraud et al. who compared delineations of observers with different levels of experience and demonstrated that users with less experience tend to draw smaller VOIs [40].

The comparison of the percentage differences of SUV_MAX_, SUV_MEAN_ and TLG showed that the SUV_MAX_ was the most stable feature that resulted only in a few cases in a difference larger than 0. In general, the SUV_MAX_ should not be segmentation dependent and the variability of the SUV_MAX_ is due to the inclusion of background-tissue in the tumor mask. E.g. for the segmentations of one lymphoma patient discrepancies of around 200% were observed using the manual approach. The tumor of this patient had a very large volume (MATV > 5000 mL) and was situated in the lower body close to the kidneys, three observers (two experienced and one less experienced observer) included voxels belonging to the kidney in the manual segmentation. This voxels were close but not part of the original tumor mask and were therefore not included in any other segmentation approach. Furthermore, in one melanoma patient more than 40% SUV_MAX_ differences were observed. These tumors also resulted in the lowest PPV/SE range for manual segmentations (when compared with the other segmentation methods). Since in this case the tumor was located very close to the heart, the predefined mask also included parts of the heart. In the manual segmentations, the user could exclude the heart manually, while for the other approaches small parts of the heart were still included in the VOI.

The most voted algorithm in the select-the-best approach was the SUV4 algorithm. However, it was not selected in the majority of the cases. Moreover, there was also no algorithm which was rejected in all cases. This underlines the fact that none of the predefined segmentation methods tested in this paper resulted in satisfying results for the complex tumors included in this study. This is in line with previous studies which reported the limitations of these commonly used and widely available algorithms [12,41,42].

In summary, our results suggest that two of the proposed strategies, namely the use of the gradient image (in combination with interactive threshold selection) or select-the-best workflow, led to less inter-observer variability than those seen with more conventional approaches. Therefore, the use of one of these strategies is recommended for the segmentation of large bulky tumors. For these tumors a fully automated method, which generate satisfactorily segmentations, does not exist as illustrated in the supplemental material. In some individual cases, e.g. when the tumor is placed close to another high uptake region, a manual correction might still be required and/or could be applied in combination with the proposed new delineation strategies. Moreover, the two strategies could also be used for a fast and reliable generation of a dataset of labeled images for the training of a CNN or a machine learning algorithm as these strategies allows for a fast (< 5 to 10 min) labelling of the images.

A possible limitation of this study might be the predefined order in which the approaches were performed. The increase in experience with the delineation software but also with the patient data might have an influence on segmentation quality. Since the segmentation approaches were ordered according to the level of user-interaction, this effect should be small. Furthermore, the images were also segmented in a specific order disease wise. Thus, the differences in segmentation quality could also be due to a loss of observer patience and care when performing segmentation tasks sequentially over an extended period. However, most observers split the work of one approach over several days, which should minimize this effect.

## Conclusion

In this study, we report on the inter-observer variability of four segmentation strategies/workflows for very large, heterogeneous and bulky tumors in PET images. Each of these workflows has a different level of user-interaction. In particular, this study included two new strategies especially implemented for large and heterogeneous tumors. These strategies provided the observer with either gradient image information (in combination with interactive threshold setting) or several predefined segmentations. Our results suggest that for these complex tumors, for every tumor type a separate validation on the most stable segmentation method should be done as none of the methods led to good results in all cases. However, the use of either gradient based or select-the-best strategy outperformed the other approaches. Hence, one of these two strategies seems preferable for bulky tumors for which segmentations always require user supervision/interaction.

## Supporting information

S1 Material Results of automatic segmentation algorithm applied on the dataset. (DOCX)

S1 Fig Segmentation results of automatic segmentation algorithm for lung cancer patients. (DOCX)

S2 Fig Segmentation results of automatic segmentation algorithm for lymphoma patients. (DOCX)

S3 Fig Segmentation results of automatic segmentation algorithm for melanoma patients. (DOCX)

S4 Fig Segmentation results of automatic segmentation algorithm for sarcoma patients. (DOCX)

S5 Fig CT image (left) and PET image (right) with predefined mask as they were presented to the user before the start of the segmentation. (DOCX)

S1 Table Lists injected activity, patient weight and times between injection and start of the scan for every patient. (DOCX)

S2 Table Lists 1^st^ and 3^rd^ quartile values, median and IQR of JC index (left) and percentage MATV differences (right) for the four approaches. (DOCX)

S3 Table Median and IQR values for percentage feature differences between performed segmentations and MV reference standard. (DOCX)
