# Supplementary material for: PET segmentation of bulky tumors: Strategies and workflows to improve inter-observer variability
Source: PLoS One. 2020 Mar 30;15(3):e0230901. doi: 10.1371/journal.pone.0230901 (PMC7105134; doi:10.1371/journal.pone.0230901)
Supplement: S1 Material — (DOCX) [file pone.0230901.s001.docx]

**Results of automatic segmentation algorithm applied on the dataset**

For illustration, we applied three automatic segmentation algorithm on the dataset of our study. All results are displayed below. We included a watershed algorithm, a region growing, and a thresholding approach. Before applying the algorithm, a bounding box was drawn around each tumor. The segmentation results of the algorithms are displayed in red. If the algorithm segmented the whole bounding box, the whole box is displayed in red. If no voxel on the displayed image slice was segmented, only the tumor is displayed.
