# Supplementary material for: PET segmentation of bulky tumors: Strategies and workflows to improve inter-observer variability
Source: PLoS One. 2020 Mar 30;15(3):e0230901. doi: 10.1371/journal.pone.0230901 (PMC7105134; doi:10.1371/journal.pone.0230901)

S3 Fig: Segmentation results of automatic segmentation algorithm for melanoma patients

**c) Melanoma patients**

**
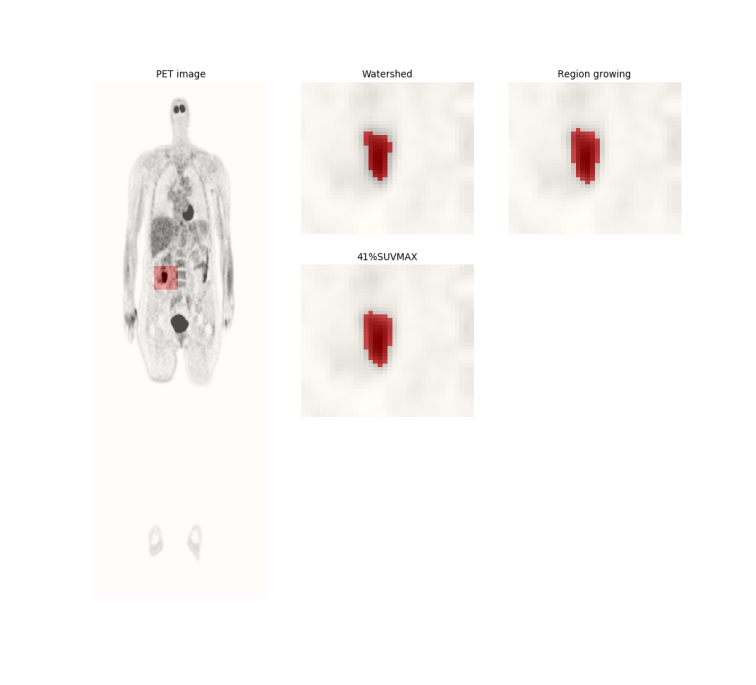

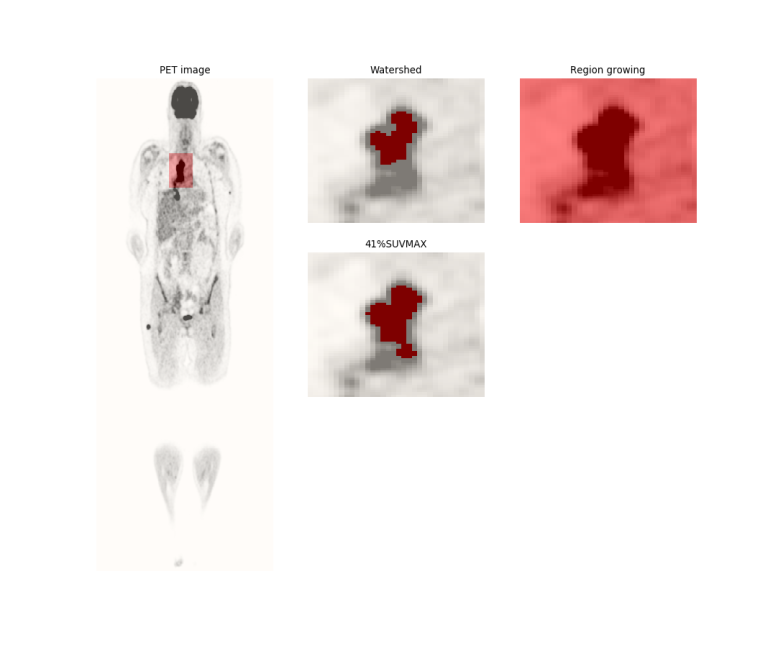
**


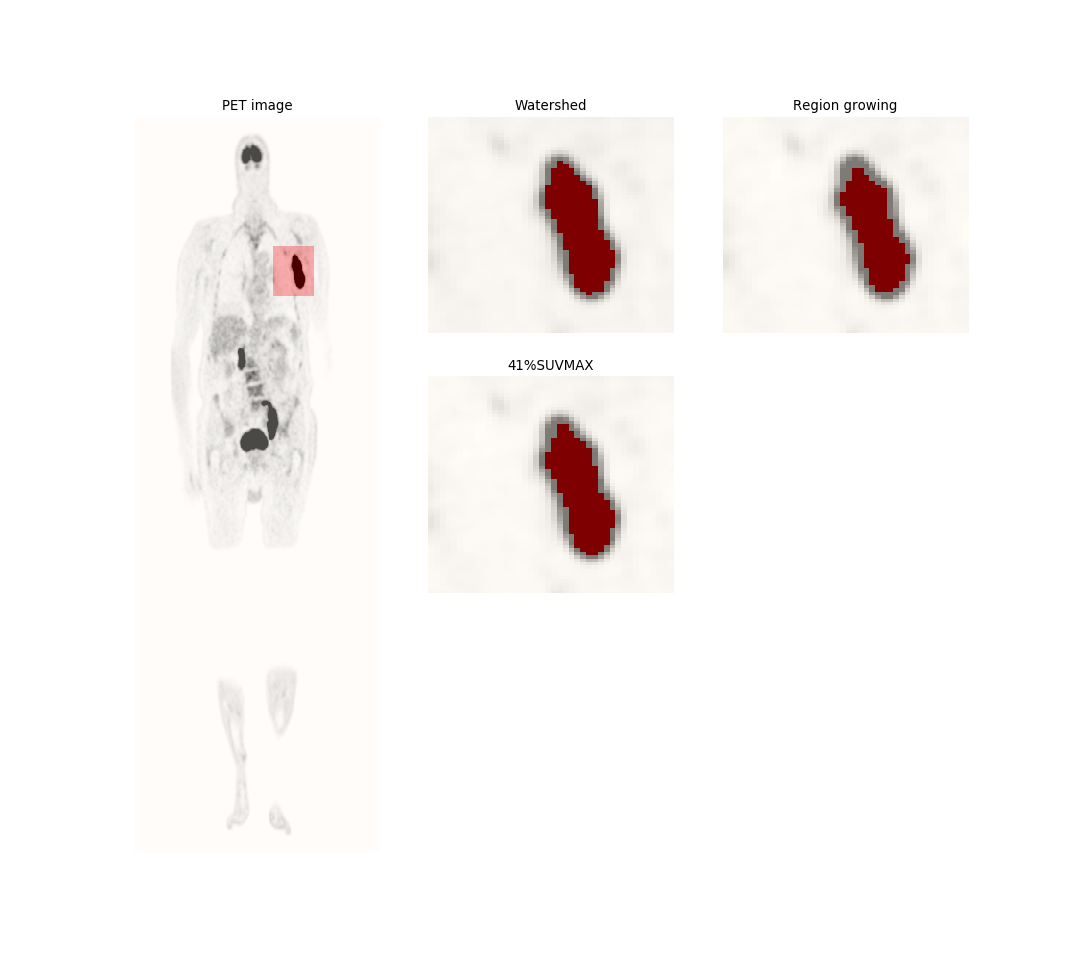

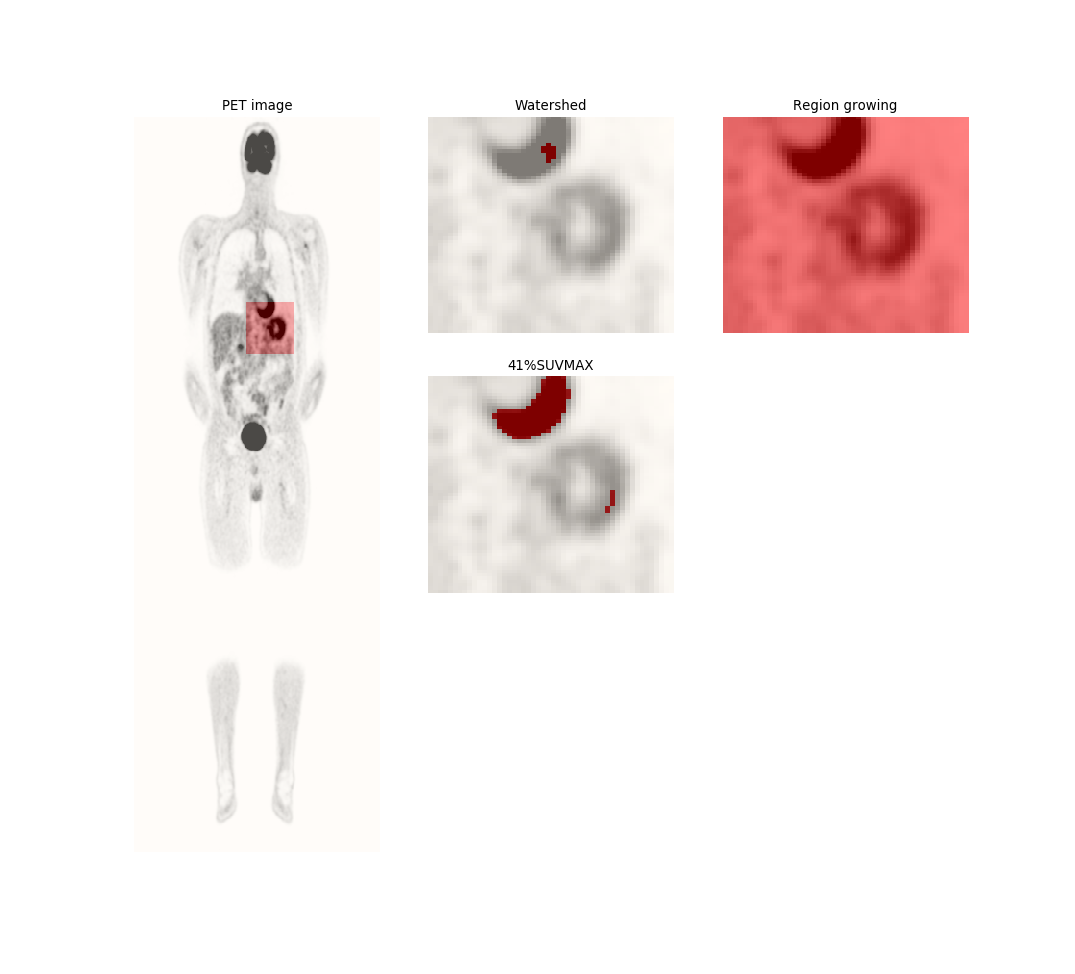


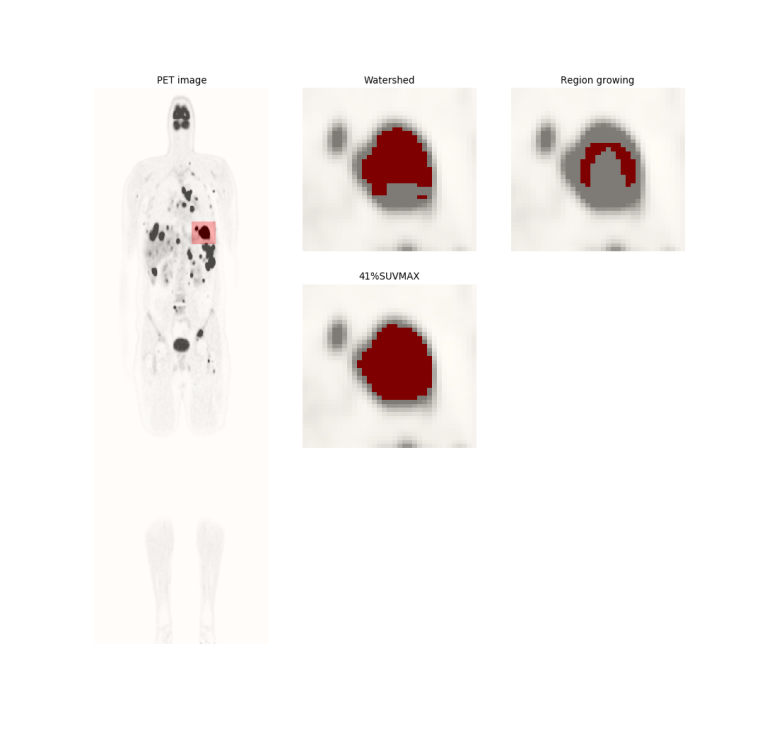

Supplement: S3 Fig — (DOCX) [file pone.0230901.s004.docx]
