# Supplementary material for: PET segmentation of bulky tumors: Strategies and workflows to improve inter-observer variability
Source: PLoS One. 2020 Mar 30;15(3):e0230901. doi: 10.1371/journal.pone.0230901 (PMC7105134; doi:10.1371/journal.pone.0230901)
Supplement: S5 Fig — (DOCX) [file pone.0230901.s006.docx]

S5 Fig: CT image (left) and PET image (right) with predefined mask as they were presented to the user before the start of the segmentation

**
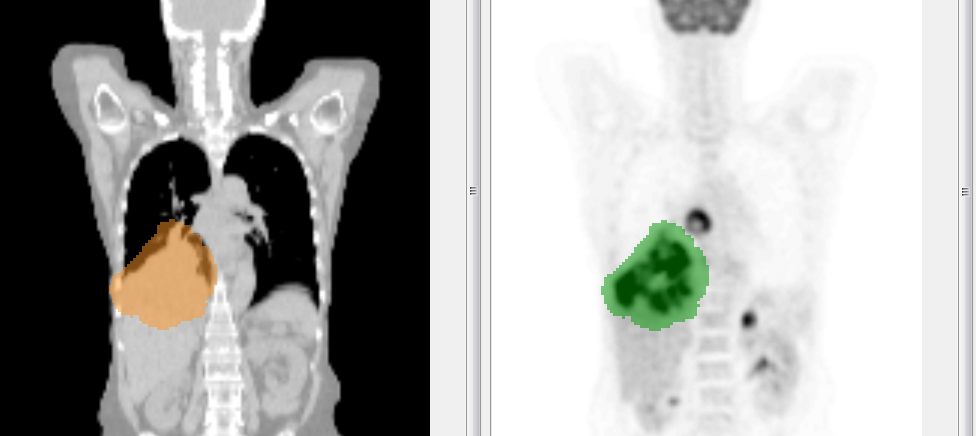
**
