# Supplementary material for: PET segmentation of bulky tumors: Strategies and workflows to improve inter-observer variability
Source: PLoS One. 2020 Mar 30;15(3):e0230901. doi: 10.1371/journal.pone.0230901 (PMC7105134; doi:10.1371/journal.pone.0230901)
Supplement: S1 Table — (DOCX) [file pone.0230901.s007.docx]

S1 Table lists injected activity, patient weight and times between injection and start of the scan for every patient

|  | Injected activity/MBq | Min. to scan | Patient weight/kg |
| --- | --- | --- | --- |
| Lung1 | 258 | 60 | 76.4 |
| Lung2 | 376 | 46 | 110 |
| Lung3 | 205 | 57 | 63 |
| Lung4 | 243 | 57 | 68 |
| Lung5 | 194 | 52 | 57 |
| Lympho1 | 115 | 61 | 65 |
| Lympho2 | 210 | 86 | 70 |
| Lympho3 | 205 | 65 | 103 |
| Lympho4 | 481 | 70 | 102 |
| Lympho5 | 388 | 66 | 67 |
| Mela1 | 389 | 58 | 73.5 |
| Mela2 | 245 | 85 | 85 |
| Mela3 | 229 | 60 | 82 |
| Mela4 | 240 | 57 | 74 |
| Mela5 | 360 | 60 | 118 |
| Sarco1 | 150 | 57 | 45 |
| Sarco2 | 159 | 54 | 73 |
| Sarco3 | 279 | 63 | 86 |
| Sarco4 | 373 | 72 | 124 |
| Sarco5 | 240 | 72 | 90 |
