# Supplementary material for: PET segmentation of bulky tumors: Strategies and workflows to improve inter-observer variability
Source: PLoS One. 2020 Mar 30;15(3):e0230901. doi: 10.1371/journal.pone.0230901 (PMC7105134; doi:10.1371/journal.pone.0230901)
Supplement: S2 Table — (DOCX) [file pone.0230901.s008.docx]

S2 Table lists 1^st^ and 3^rd^ quartile values, median and IQR of JC index (left) and percentage MATV differences (right) for the four approaches

| Segmentation method | 1st quartile JC | Median JC | 3rd quartile JC | IQR JC | 1st quartile %MATV diff. | Median %MATV diff. | 3rd quartile %MATV diff. | IQR %MATV diff. |
| --- | --- | --- | --- | --- | --- | --- | --- | --- |
| Select-the-best | 0.69 | 0.87 | 1 | 0.31 | -14% | 0% | 15% | 29% |
| Gradient | 0.72 | 0.83 | 0.92 | 0.2 | -16% | 1% | 19% | 35% |
| Threshold | 0.64 | 0.8 | 0.91 | 0.27 | -16% | 4.2% | 27% | 43% |
| Manual | 0.63 | 0.74 | 0.8 | 0.17 | -12% | 11.5% | 28% | 40% |
