# Supplementary material for: PET segmentation of bulky tumors: Strategies and workflows to improve inter-observer variability
Source: PLoS One. 2020 Mar 30;15(3):e0230901. doi: 10.1371/journal.pone.0230901 (PMC7105134; doi:10.1371/journal.pone.0230901)
Supplement: S3 Table — (DOCX) [file pone.0230901.s009.docx]

S3 Table : median and IQR values for percentage feature differences between performed segmentations and MV reference standard

|  | MATV median %diff | MATV IQR %diff | SUVMAX median %diff | SUVMAX IQR %diff | SUVMEAN median %diff | SUVMEAN IQR %diff | TLG median %diff | TLG IQR %diff |
| --- | --- | --- | --- | --- | --- | --- | --- | --- |
| Select-the-best | 0% | 30.1% | 0.15% | 0.42% | 0.24% | 11.2% | 0.29% | 15.4% |
| Gradient | -5% | 19.3% | 0.14% | 0.51% | 1.14% | 12.8% | -2.7% | 16.8% |
| Threshold | -11.1% | 31% | 0.14% | 0.47% | 4.21% | 12.1% | -5.3% | 15.1% |
| Manual | -19.2% | 40.5% | 0.14% | 0.53% | 7.4% | 17.9% | -8.7% | 18.9% |
